# The association between normal serum sodium levels and bone turnover in patients with type 2 diabetes

**DOI:** 10.3389/fendo.2022.927223

**Published:** 2022-10-27

**Authors:** Hai-yan Huang, Zhi-qi Huang, Ling-yan Hua, Wang-shu Liu, Feng Xu, Xiao-qin Ge, Chun-feng Lu, Jian-bin Su, Xue-qin Wang

**Affiliations:** ^1^ Department of Endocrinology, Affiliated Hospital 2 of Nantong University and First People’s Hospital of Nantong City, Nantong, China; ^2^ Department of General Surgery, Affiliated Hospital 2 of Nantong University and First People’s Hospital of Nantong City, Nantong, China; ^3^ Department of Ophthalmology, Affiliated Hospital 2 of Nantong University and First People’s Hospital of Nantong City, Nantong, China

**Keywords:** type 2 diabetes, bone turnover, bone formation, bone resorption, sodium, bone mineral density

## Abstract

**Background:**

Sodium is a critically important component of bones, and hyponatremia has firmly been established as a risk factor associated with the incidence of fragility fractures. However, researches have also revealed that lower serum sodium are linked to reductions in muscle mass and a higher risk of cardiovascular disease even when these levels are within the normal range. Accordingly, this study was developed to examine the relationships between normal serum sodium concentrations and bone turnover in patients with type 2 diabetes (T2D).

**Methods:**

Patients with T2D were enrolled in the present study from January 2021 to April 2022. All patients underwent analyses of serum sodium levels, oral glucose tolerance testing (OGTT), bone turnover markers (BTMs), and dual-energy X-ray absorptiometry (DXA) scanning. BTMs included bone formation markers osteocalcin (OC) and N-terminal propeptide of type-I procollagen (PINP), and bone resorption marker C-terminal telopeptide (CTx). Patients were stratified into three subgroups based on the tertiles of their serum sodium concentrations.

**Results:**

In total, 372 patients with T2D and sodium levels in the normal range were enrolled in this study. Serum OC and PINP levels were increased from subgroup with the low sodium tertile to that with the high sodium tertile (*p* for trend < 0.05), whereas CTx level was comparable among the subgroups. A positive correlation was detected between serum sodium levels and both lnOC (*r* = 0.210, *p* < 0.001) and lnPINP (*r* = 0.196, *p* < 0.001), with these relationships remaining significant even following adjustment for age, sex, body mass index (BMI), and HbA1c. Only after adjusting for these four factors a positive correlation was detected between serum sodium levels and CTx levels (*r* = 0.108, *p* < 0.05). Linear regression analyses revealed that following adjustment for potential covariates, serum sodium level was and positively significantly associated with lnOC level (*β* = 0.134, *t* = 2.281, *p* < 0.05) and PINP level (*β* = 0.179, *t* = 3.023, *p* < 0.01).

**Conclusion:**

These results highlight a significant association between low-normal serum sodium levels and low bone turnover.

## Introduction

Fragility fractures are a common complication in patients with type 2 diabetes (T2D), contributing to high rates of morbidity and mortality together with mounting public health costs (1). Relative to matched populations without T2D, individuals affected by this metabolic disease may exhibit normal or slightly increased bone mineral density (BMD) such that this parameter may not effectively reflect the risk of fragility fractures in this patient cohort (2). Bone turnover is a continuous process critical for bone health that entails both the resorption and formation of bone such that old, worn bone tissue is replaced with a new calcified matrix (3). This turnover process can be noninvasively and repeatedly monitored through the assessment of bone biopsy-validated bone turnover markers (BTMs) (4). Several reports have documented significant reductions in these BTMs levels in patients with T2D relative to matched populations unaffected by T2D (5–7). As such, further research clarifying the risk factors associated with low bone turnover in patients with T2D and efforts to facilitate appropriate interventions represent a critical component of T2D management.

Sodium is an essential element for normal physiological processes, and patients with T2D may experience osmotic diuresis as a consequence of disease-related hyperglycemia, contributing to the excess excretion of sodium in the urine and resulting in hyponatremia (8). Such hyponatremia is associated with a range of adverse clinical findings and pathophysiological changes in patients with T2D (9). Sodium is an abundant mineral in the bone, wherein roughly 40% of sodium can be rapidly exchanged with sodium in circulation (10). As a consequence, bone-derived sodium can enter the bloodstream in individuals suffering from hyponatremia, thereby sustaining blood pressure, blood volume, and tissue perfusion while potentially also inducing some level of bone resorption (11). Significantly higher hyponatremia rates have been observed in individuals with fractures relative to those without fractures in a case-control study (12). Both hyponatremia and hypernatremia significantly increased the short-term risk of death in patients with hip fracture (13). Data derived from the NHANES database further indicated that individuals exhibiting chronic mild hyponatremia were significantly more likely to suffer from hip osteoporosis (14). Tibial biopsy samples collected from hyponatremia model animals also revealed imbalanced bone turnover attributable to higher levels of osteoclasts without any corresponding change in osteoblasts (14). Prior studies have primarily explored the association between hyponatremia and bone health, whereas there have been few analyses examining how bone turnover relates to serum sodium levels within the normal range. In females with anorexia nervosa, serum sodium levels on the low end of the normal range (low-normal level) were closely related to bone loss (15). Given that most patients with T2D exhibit serum sodium levels within this normal range, there is a clear need for further studies of the relationship between these levels and bone turnover in this patient population. This study was conducted based on the hypothesis that normal sodium levels are closely related to BTMs in patients with T2D, with individuals exhibiting low-normal sodium levels being at greater risk of experiencing suppressed bone turnover as compared to individuals with high-normal sodium levels.

This cross-sectional observational study was designed to examine the association between normal serum sodium levels and BTMs in patients with T2D.

## Methods

### Study design and participants

**Figure 1:**
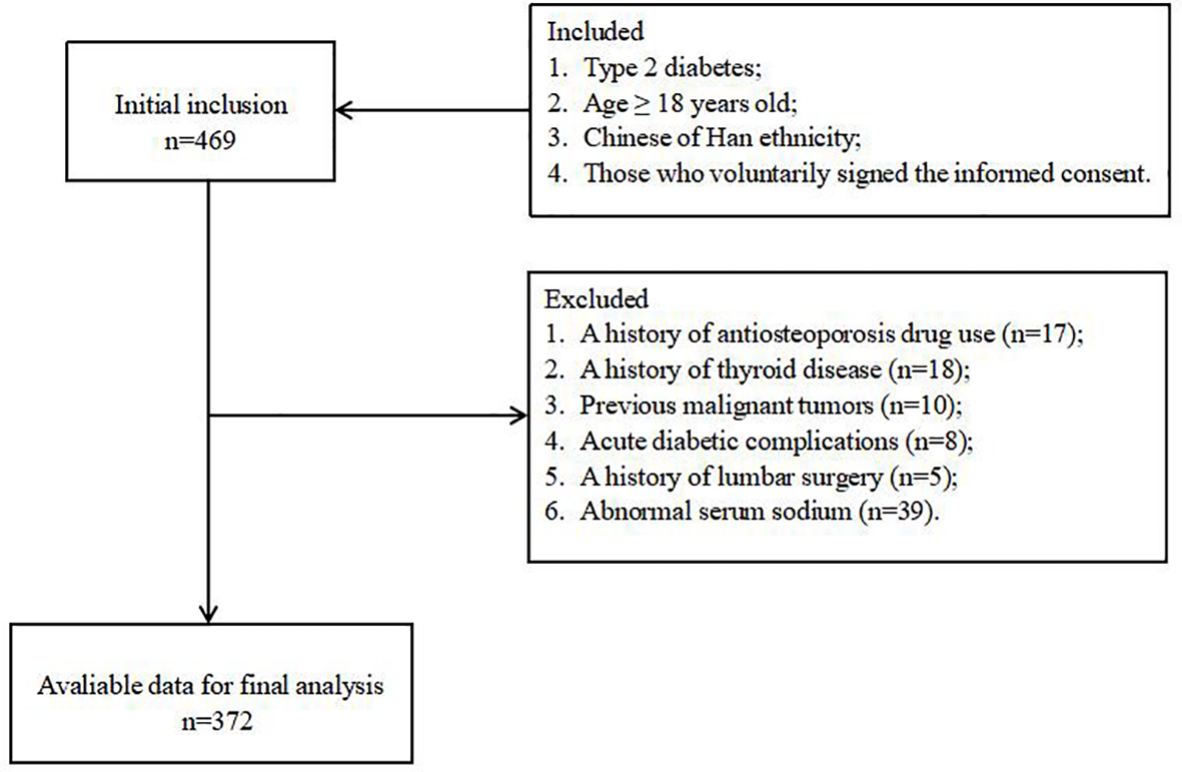
The study flowchart.

This cross-sectional observational study enrolled individuals with T2D who were admitted to the Department of Endocrinology of the Second Affiliated Hospital of Nantong University between January 2021 and April 2022. The study flowchart is shown in **Figure 1**. The inclusion criteria were as follows: (1) diagnosed with T2D based on the criteria published by the American Diabetes Association in 2013 (16); (2) age ≥ 18 years old; (3) Chinese of Han ethnicity; (4) those who voluntarily signed the informed consent. Patients were excluded from the study if they had type 1 diabetes, a history of steroid or antiosteoporosis drug use (e.g., vitamin D, calcium tablet, bisphosphonates, denosumab and selective estrogen receptor), a history of current or prior antiandrogen therapy, malignant tumors, chronic hepatitis, heart failure, pituitary or adrenal gland diseases, acute diabetic complications (including severe hypoglycemic, diabetic ketoacidosis, hyperosmolar hyperglycemic state, and diabetic lactic acidosis), a history of lumbar surgery, a history of thyroid or parathyroid disease, or a recent history of sodium supplementation. Based on these criteria we included a total of 417 patients with T2D, 9 of whom had hyponatremia (< 135mmol/L), 372 of whom had normal serum sodium levels, and 30 of whom had hypernatremia (> 145mmol/L). The 372 patients with normal serum sodium levels were ultimately included in this study.

### Basic data collection

All study participants were evaluated by a clinician using a questionnaire designed to gather clinical details including demographic factors, history of medication use, lifestyle factors, and history of diseases. Upon enrollment, all patients underwent physical examination, including analyses of body mass index (BMI = weight/height^2^) and blood pressure measured as the average of three recordings made using a standard mercury sphygmomanometer.

### Laboratory examination

Following study enrolment, fasting blood samples were collected from all participants for hematological testing, while fresh first-void morning urine samples were collected to measure urinary creatinine and albumin levels, with the urinary albumin to urinary creatinine ratio (UACR) thereby being calculated. All analyses of renal function and lipid indices were performed using an automated biochemical analyzer (Model 7600, Hitachi), and measured inter- and intra-assay coefficients of variation (CVs) were < 2.8%. Estimated glomerular filtration rate (eGFR) values were calculated using the CKD-EPI creatinine-cystatin C equation (17). HbA1c levels were measured *via* ion exchange-based high-performance liquid chromatography (HPLC) with a hemoglobin analysis system (D-10, Bio-Rad), with inter- and intra-assay CVs < 3.0%. Chemiluminescent measurements of serum OC, CTx, PINP, 25-hydroxyvitamin D (25(OH)D), and parathyroid hormone (PTH) levels were measured with an automated immunoassay system (iSYS, Immunodiagnostic Systems Ltd., Boldon), with respective CVs that were below 2.0%, 5.4%, 4.5%, 4.0%, and 4.5% and 4.0%.

### Oral glucose tolerance test and evaluation of insulin sensitivity and islet β-cell function

Before conducting OGTT, the serum glucose should be controlled stably and hypoglycemic therapy should be discontinued for a minimum of 24h. Following an overnight fasting, an OGTT was performed by providing patients with 75 g anhydrous glucose, with venous blood samples then being collected after 0, 30, 60, 120, and 180 minutes to simultaneously measure serum concentrations of glucose and C-peptide. The effects of exogenous insulin were eliminated by calculating β-cell secretion and insulin resistance indices based on C-peptide levels. The trapezoidal principle for reflection β-cell secretion was used to calculate the C-peptide area under the curve (AUCCP) (18), and insulin resistance was measured based on the HOMA-IRCP, which was calculated as (fasting glucose × fasting C-peptide)/22.5 (19).

### Measurement of bone mineral density

BMD was measured *via* lumbar spine (L1-L4) and total hip dual-energy X-ray absorptiometry (DXA) scans conducted with a Prodigy Scanner (GE-Healthcare, Madison), with lumbar spine and total hip T-scores being measured for subsequent analysis. T-score calculations were performed with reference to the peak BMD of healthy gender- and ethnicity-matched young adults. DXA CVs remained below 0.24% in daily quality control analyses. Patients exhibiting hip or spine T-scores ≤ -2.5 were diagnosed with osteoporosis.

### Statistical analyses

The overall patient cohort was stratified into three subgroups according to the tertiles of serum sodium level: T1 (135.0 – 140.3 mmol/L), T2 (140.4 – 142.4 mmol/L), and T3 (142.5 – 145.0 mmol/L). Continuous data were analyzed with the Kolmogorov-Smirnov test to determine whether they conformed to a normal distribution. OC, CTx, and PINP levels were then subject to natural logarithmic transformation to normalize these distributions for subsequent analyses. Continuous data that were and were not normally distributed and categorical variables were respectively reported as means ± SD, medians (25% and 75% quartiles), and frequencies (percentages), with these three respective data types being compared *via* one-way ANOVAs, Kruskal-Wallis tests, and chi-square tests. Correlative relationships between serum sodium levels and clinical variables were assessed with Spearman’s bivariate correlation analyses, while relationships between serum sodium levels and BTMs were evaluated using Pearson’s bivariate correlation analyses and partial correlation analyses. As BTM levels may be impacted by age, gender, BMI, and HbA1c levels, three partial correlation analyses were used to explore the associations between serum sodium levels and BTMs when adjusting for these parameters. Spearman correlation analyses were additionally employed to examine the associations between serum sodium levels and BTMs in elderly and non-elderly patients with T2D and in male and female patients with T2D, respectively. Multivariate linear regression analyses were then used to examine the independent association of serum sodium levels with the BTMs levels, correcting for possible confounding variables. Data were analyzed with SPSS 18.0 (IBM SPSS Inc., USA), and P < 0.05 was the threshold of significance.

## Results

### Patient characteristics

The clinical characteristics of the patients included in the overall study population and in the three serum sodium level-based subgroups are summarized in **Table 1**. Significant differences in male proportions, metformin use, HbA1c levels and total cholesterol (TC) levels were observed among these three patient subgroups (all *p <* 0.05). No differences were observed among these groups with respect to age, duration of T2D, systolic blood pressure (SBP), diastolic blood pressure (DBP), BMI, history of smoking, statin use, use of other antidiabetic treatments, use of antihypertensive treatments, AUC_CP_, HOMA-IR_CP_, serum uric acid (UA) levels, cystatin C levels, eGFR, UACR, triglyceride (TG) levels, high-density lipoprotein cholesterol (HDL-C) levels, LDL-C levels, PTH levels, 25(OH)D levels, lumbar spine, and total hip BMDs, or the proportions of patients diagnosed with osteoporosis (all *p* > 0.05). Significant correlations were also detected between serum sodium levels and SBP, AUC_CP_, UACR, and TG levels (all *p <* 0.05) using Spearman’s bivariate correlation analyses (**Supplementary Table 1**).

**Table 1:** Patient characteristics.

Variables	Total	T1	T2	T3	*p* value
*n*	372	126	129	117	
Sodium range (mmol/L)	135.0-145.0	135.0-140.3	140.4-142.4	142.5-145.0	
Sodium (mmol/L)	141.17 ± 2.25	138.62 ± 1.40	141.46 ± 0.61	143.59 ± 0.75	
Age (years)	58.56 ± 12.80	57.56 ± 13.84	58.95 ± 12.75	59.21 ± 11.68	0.555
Male, *n* (%)	202(54.3)	78(61.9)	60(46.5)	64(54.7)	0.047
Diabetic duration (years)	6.0(2.0-10.0)	5.0(2.0-10.0)	7.0(2.0-10.0)	8.0(2.0-12.5)	0.179
BMI (kg/m^2^)	25.74 ± 3.83	26.11 ± 4.27	25.32 ± 3.59	25.82 ± 3.59	0.263
SBP (mmHg)	136.84 ± 19.18	134.60 ± 19.52	137.30 ± 19.05	138.73 ± 18.89	0.233
DBP (mmHg)	83.38 ± 10.58	83.45 ± 12.23	83.41 ± 10.59	83.28 ± 10.42	0.992
Smoking history, *n* (%)	36(9.7)	9(7.1)	14(10.9)	12(10.3)	0.556
Antidiabetic treatments					
Insulin treatment, *n* (%)	110(29.6)	34(27.0)	35(27.1)	41(35.0)	0.293
Metformin, *n* (%)	183(49.2)	62(49.2)	75(58.1)	46(39.3)	0.013
Acarbose, *n* (%)	30(8.1)	9(7.1)	7(5.4)	14(12.0)	0.153
Insulin-secretagogues, *n* (%)	123(33.1)	36(28.6)	41(31.8)	46(39.3)	0.191
Insulin-sensitizers, *n* (%)	40(10.8)	14(11.1)	16(12.4)	10(8.5)	0.614
DPP-4 inhibitors, *n* (%)	30(8.1)	13(10.3)	7(5.4)	10(8.5)	0.348
SGLT-2 inhibitors, *n* (%)	42(11.3)	15(11.9)	13(10.1)	14(12.0)	0.865
Antihypertensive treatments					
CCB, *n* (%)	91(24.5)	38(30.2)	25(19.4)	28(23.9)	0.133
ARB, *n* (%)	82(22.0)	29(23.0)	23(17.8)	30(25.6)	0.319
β-blockers, *n* (%)	28(7.5)	10(7.9)	8(6.2)	10(8.5)	0.767
Diuretics, *n* (%)	29(7.8)	12(9.5)	7(5.4)	10(8.5)	0.767
Statins medications, *n* (%)	30(8.1)	10(7.9)	10(7.8)	10(8.5)	0.972
HbA1c (%)	9.30 ± 2.25	9.91 ± 2.31	9.00 ± 2.04	8.99 ± 1.96	<0.001
AUC_CP_	13.11(8.32-18.93)	11.60(8.50-17.72)	12.77(7.95-19.62)	13.65(9.44-19.84)	0.306
HOMA-IR_CP_	0.46(0.25-0.74)	0.47(0.25-0.78)	0.42(0.24-0.76)	0.47(0.26-0.68)	0.927
Serum UA (umol/L)	312.66 ± 107.16	330.33 ± 121.73	300.10 ± 100.61	307.39 ± 95.05	0.067
Cystatin C (mg/L)	0.96 ± 0.41	1.01 ± 0.48	0.92 ± 0.44	0.95 ± 0.30	0.263
eGFR (ml/min/1.73m^2^)	98.01 ± 26.88	96.34 ± 29.17	101.77 ± 26.97	95.7 ± 23.95	0.197
UACR (mg/g)	16.25(8.13-45.00)	18.40(8.60-58.65)	18.60(8.75-40.08)	12.90(7.00-34.40)	0.077
TG (mmol/L)	1.68(1.05-2.59)	1.81(1.19-2.96)	1.57(1.02-2.68)	1.65(1.03-2.20)	0.062
TC (mmol/L)	4.40 ± 1.26	4.66 ± 1.57	4.29 ± 1.09	4.24 ± 0.99	0.016
HDL-C (mmol/L)	1.12 ± 0.26	1.11 ± 0.28	1.13 ± 0.23	1.10 ± 0.26	0.524
LDL-C (mmol/L)	2.75 ± 0.89	2.79 ± 0.86	2.70 ± 0.94	2.75 ± 0.86	0.743
lnOC	2.41 ± 0.41	2.33 ± 0.43	2.44 ± 0.39	2.47 ± 0.39	0.014
lnCTx	-0.81 ± 0.56	-0.84 ± 0.58	-0.84 ± 0.59	-0.75 ± 0.50	0.353
lnPINP	3.65 ± 0.44	3.57 ± 0.46	3.67 ± 0.44	3.70 ± 0.40	0.037
PTH (pg/mL)	33.70(25.70-43.90)	32.80(25.40-43.13)	34.20(26.85-44.10)	34.00(25.03-45.00)	0.661
25(OH)D (ng/mL)	16.82 ± 7.19	16.01 ± 7.49	17.41 ± 7.14	17.04 ± 6.90	0.277
Lumbar spine BMD	0.96 ± 0.17	0.98 ± 0.18	0.93 ± 0.18	0.96 ± 0.15	0.149
Total hip BMD	1.03 ± 0.13	1.03 ± 0.12	1.03 ± 0.15	1.03 ± 0.12	0.964
Osteoporosis, *n* (%)	61(16.4)	15(11.9)	31(24.0)	15(12.8)	0.155

Normally distributed values in the table are given as the mean ± SD, skewed distributed values are given as the median (25 and 75% interquartiles), and categorical variables are given as frequency (percentage).

BMI, body mass index; SBP/DBP, systolic/diastolic blood pressure, Insulin-secretagogues insulin secretagogues, Insulin-sensitizers insulin sensitizing agents; DPP-4, inhibitors dipeptidyl peptidase-4 inhibitors, sodium-glucose co-transporter-2 inhibitors SGLT-2 inhibitors; CCB, calcium channel blockers; ARB, angiotensin receptor blockers; HbA1c glycosylated hemoglobin A1c; AUC_CP_, area under the C-peptide curve; HOMA-IR_CP_, homeostasis model assessment of insulin resistance using C-peptide; Serum UA, serum uric acid; eGFR, estimated glomerular filtration rate; UACR, urinary albumin-to-creatinine ratio; TG, triglyceride; TC, total cholesterol; HDL-C, high-density lipoprotein cholesterol; LDL-C, low-density lipoprotein cholesterol; OC, osteocalcin; CTx, C-terminal telopeptide; PINP, N-terminal propeptide of type-I procollagen; PTH, parathyroid hormone; BMD, bone mineral density.

### Associations between serum sodium levels and BTMs

**Figure 2:**
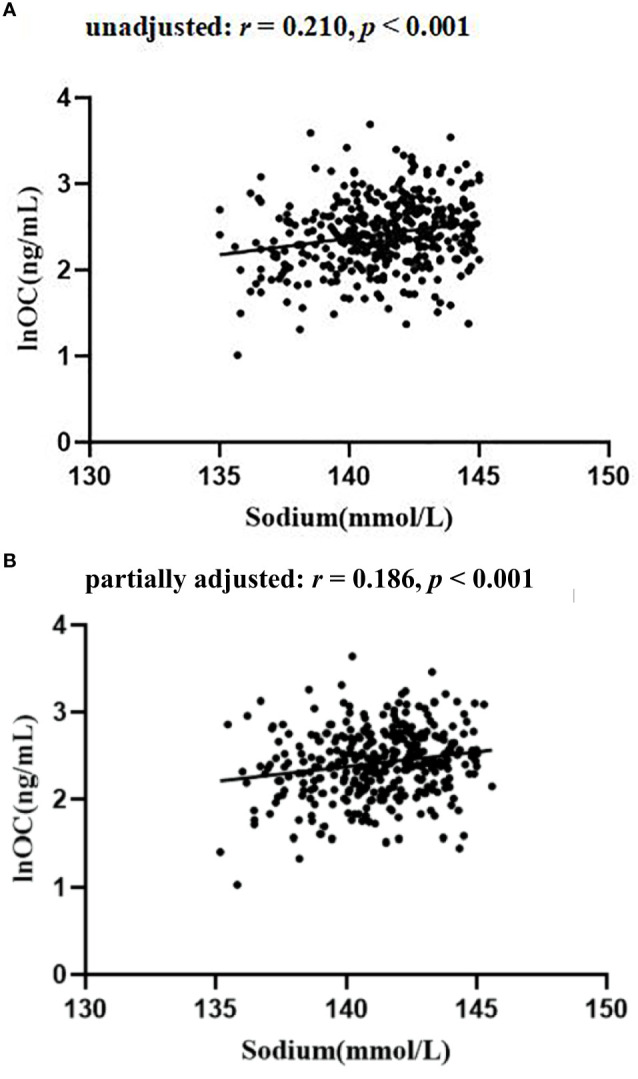
The relationship between serum sodium and lnOC in patients with T2D **(A)** unadjusted; **(B)** partially adjusted for age, sex, BMI and HbA1 c.

**Figure 3:**
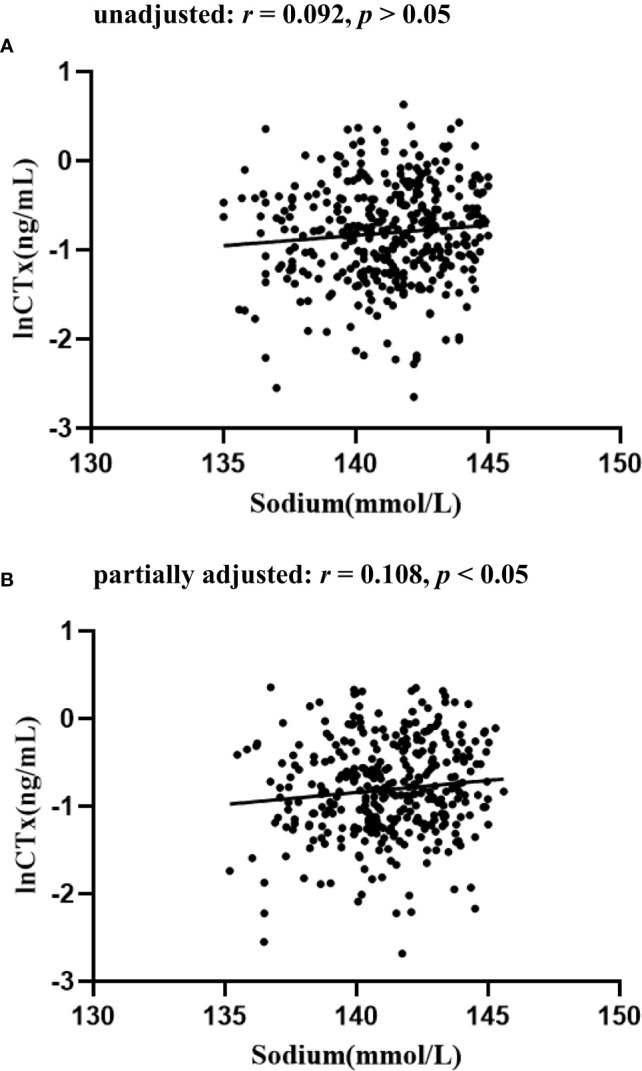
The relationship between serum sodium and lnCTx in patients with T2D **(A)** unadjusted; **(B)** partially adjusted for age, sex, BMI and HbA1 c.

**Figure 4:**
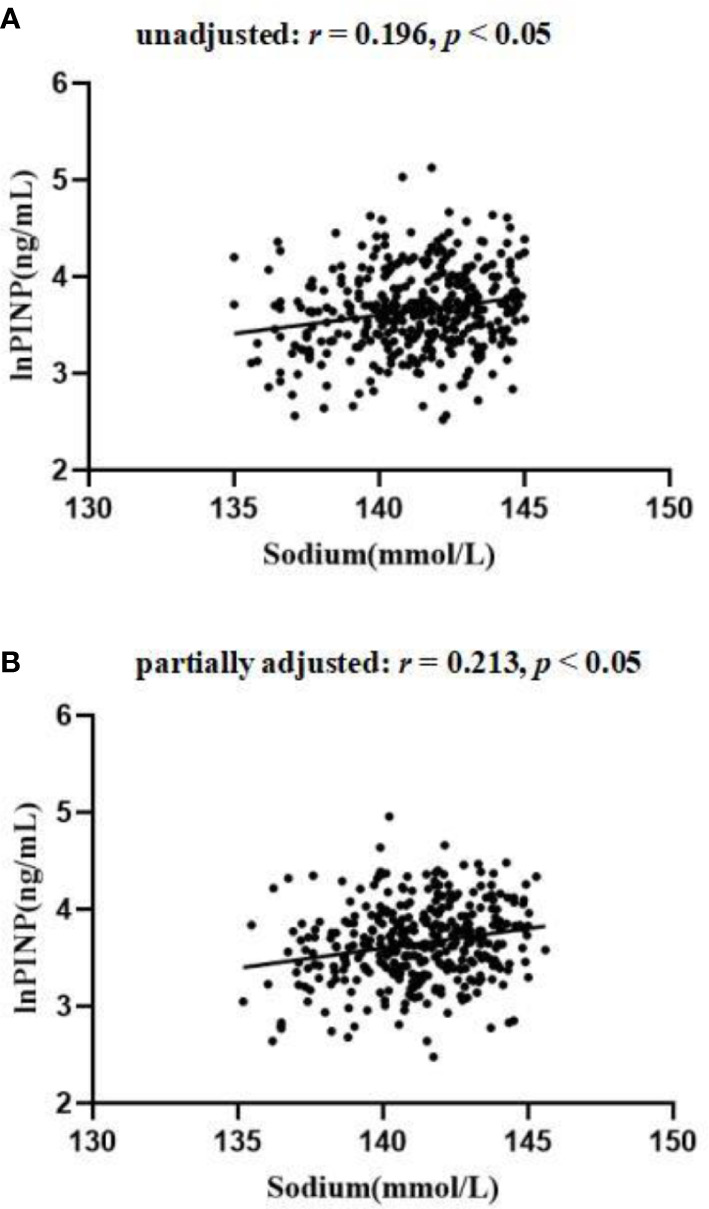
The relationship between serum sodium and lnPINP in patients with T2D **(A)** unadjusted; **(B)** partially adjusted for age, sex, BMI and HbA1c.

Significant differences in lnOC and lnPINP levels were detected among these three serum sodium level-based patient subgroups (all *p <* 0.05), while no differences in lnCTx were detected (*p* > 0.05) (**Table 1**). Significant positive correlations were detected between serum sodium levels and both lnOC and lnPINP even after adjusting for patient age, sex, BMI, and HbA1c levels (*p <* 0.05; **Table 2**; **Figures 2**–**4**). A significant positive correlation between serum sodium levels and lnCTx was only observed after adjustment for age, sex, BMI, and HbA1c levels (*p* < 0.05). As serum sodium concentrations can be impacted by patient age and sex, Spearman’s bivariate correlation analyses were conducted to examine the association between serum sodium levels and BTMs in elderly and non-elderly patients and in male and female patients (**Supplementary Table 2**), revealing results consistent with those in the overall patient cohort.

**Table 2:** Associations between serum sodium levels and BTMs.

Variables	Unadjusted		Adjusted	
	*r*	*p* value	*r*	*p* value
lnOC	0.210	<0.001	0.186	<0.001
lnCTx	0.092	0.076	0.108	0.044
lnPINP	0.196	<0.001	0.213	<0.001

r Pearson’s correlation coefficient.

Adjusted for age, sex, BMI and HbA1c.

### Independent associations of serum sodium level with BTMs levels

As shown in **Table 3**, serum sodium level was significantly and positively associated with lnOC level (*β* = 0.210, *t* = 4.141, *p* < 0.001, *R^2^* = 0.044) and lnPINP level (*β* = 0.196, *t* = 3.854, *p* < 0.001, *R^2^* = 0.039), while there was no significant association between serum sodium level and lnCTx level (*p* > 0.05). After adjusting possible clinical covariates in each model step by step, the *R^2^* was gradually increased. In the fully adjusted model 3, serum sodium level was still significantly and positively associated with (*β* = 0.134, *t* = 2.281, *p* < 0.05, *R^2^* = 0.227) and lnPINP level (*β* = 0.179, *t* = 3.023, *p* < 0.01, *R^2^* = 0.215), while there was still no significant association between serum sodium level and lnCTx level (*p* > 0.05).

**Table 3:** Independent associations of serum sodium level with BTMs levels *via* multivariate linear regression analysis..

Models	B (95% CI)	*β*	*t*	*p*	*R^2^* for model
OC					
Model 0	0.038(0.020-0.056)	0.210	4.141	<0.001	0.044
Model 1	0.040(0.022-0.059)	0.222	4.249	<0.001	0.086
Model 2	0.037(0.019-0.055)	0.204	3.957	<0.001	0.169
Model 3	0.025(0.003-0.047)	0.134	2.281	0.023	0.227
CTx					
Model 0	0.023(-0.002-0.058)	0.092	1.777	0.076	0.008
Model 1	0.031(0.006-0.057)	0.124	2.391	0.017	0.096
Model 2	0.031(0.005-0.058)	0.125	2.362	0.019	0.128
Model 3	0.019(-0.011-0.050)	0.074	1.247	0.213	0.212
PINP					
Model 0	0.038(0.019-0.058)	0.196	3.854	<0.001	0.039
Model 1	0.043(0.024-0.063)	0.224	4.409	<0.001	0.133
Model 2	0.041(0.022-0.061)	0.214	4.150	<0.001	0.169
Model 3	0.036(0.013-0.059)	0.179	3.023	0.003	0.215

Model 0: unadjusted model.

Model 1: adjusted for age, sex, diabetic duration, BMI, SBP, DBP, smoking history.

Model 2: additionally adjusted for antidiabetic treatments, antihypertensive treatments, statins medications.

Model 3: additionally adjusted for HbA1c, eGFR, TG, TC, HDL-C, LDL-C.

## Discussion

This cross-sectional observational study was developed to explore the associations between serum sodium levels and BTMs in patients with T2D. These analyses revealed that serum OC and PINP levels differed significantly among the three subgroups, whereas the same was not true for CTx levels. Serum sodium levels were also positively correlated with lnOC and lnPINP values following adjustment for patient age, gender, BMI, and HbA1c levels. Moreover, after adjusting possible covariates *via* multiple linear regression analysis, serum sodium level was still significantly and positively associated with serum OC and PINP level. Together, these results suggest a potential role for low-normal serum sodium levels in decreased bone turnover in individuals with T2D.

No prior studies to our knowledge have specifically focused on the association between serum sodium levels within the normal range and bone turnover in individuals with T2D. There have been a range of studies exploring the relationship between bone health and hyponatremia in individuals with subarachnoid hemorrhage (4), inappropriate antidiuresis syndrome patients (20), patients treated with antiepileptic drugs (21), elderly populations (22), and animal model systems (23). Of the 411 patients with T2D initially enrolled in this study, just 2.2% (9/411) exhibited hyponatremia, underscoring the need to focus on how variations in normal serum sodium levels relate to bone turnover in patients with T2D. Here, serum sodium levels were found to be significantly positively correlated with the BTMs OC and PINP, while they were unrelated to CTx levels. Consistently, subarachnoid hemorrhage patients that developed acute mild hyponatremia additionally exhibited significant reductions in bone formation but not bone resorption (4). Two factors may explain the lack of any observed correlation between sodium and CTx levels. For one, low bone turnover in patients with T2D is primarily a consequence of reduced bone formation (24). Moreover, the mechanisms through which hyponatremia can promote bone resorption detailed in prior studies largely center around the mobilization of bone sodium stores to maintain circulating sodium concentrations (11), whereas this effect may be less pronounced when sodium levels are within the normal range.

Hyperglycemia is a pathological condition associated with myriad complications in patients with T2D (25). Sodium levels and glycemic control exhibit a bidirectional regulatory relationship. In healthy males, insulin sensitivity was significantly reduced following the moderate restriction of sodium intake for 5 days (26). The restriction of sodium intake has also been reported to increase renin and aldosterone levels while significantly reducing the secretion of insulin and C-peptide following acute glycemic stimulation, albeit without any corresponding impact on insulin sensitivity (27). Serum sodium levels may thus be closely associated with insulin deficiencies and hyperglycemia in patients with T2D. Accordingly, a significant association was herein observed between serum sodium levels, HbA1c, and insulin deficiency (as measured by a reduction in AUC_CP_) in patients with T2D. Another cross-sectional study found that BTMs in patients with T2D with an HbA1c > 7% were significantly reduced (28). In a study enrolling 5,277 patients with T2D, Guo et al. determined that β-cell function (as measured based on HOMA-%β) was positively correlated with BTM levels (29). Here, serum sodium levels were found to be independently related to BTMs even following adjustment for glycemic control. In addition, significant negative correlations were also observed in the present study between serum sodium levels and TG levels and TC levels. Consistently, a review published in 2017 concluded that low sodium intake for 2 weeks could significantly increased serum TG and TC levels (30). This suggests that impaired glycemic control, insulin deficiency and abnormal lipid profiles may partially explain the observed relationship between low-normal serum sodium levels and low bone turnover in patients with T2D.

Bone is a highly vascularized organ such that changes in bone blood supply can suppress bone turnover (31). Biopsy samples have revealed evidence of arteriosclerosis in intraosseous arterioles, suggesting that arteriosclerosis may play a key role in the incidence of suppressed bone turnover in patients with T2D (32). Here, a significant negative correlation between serum sodium levels and both TG and HbA1c levels was detected, with both of these factors being closely associated with atherosclerotic risk (33). Hou et al. similarly performed a cross-sectional analysis of Chinese individuals without diabetes, revealing rising serum LDL-C levels in individuals with low serum sodium levels over an average 5.5-year follow-up period (34). In an 11-year follow-up study, older men exhibiting low-normal serum sodium levels experienced an increased risk of cardiovascular events and cardiac death as compared to individuals with serum sodium levels on the high end of the normal range (35). Serum sodium levels were also significantly negatively correlated with UACR levels, which are frequently used to monitor diabetic kidney disease and as a surrogate biomarker for systemic microcirculatory injury (36). As such, low-normal serum sodium levels may play a role in promoting arteriosclerosis, contributing to the suppression of bone turnover.

Both inflammation and oxidative stress are also important factors that can contribute to decreased bone turnover in patients with T2D (37). Signaling *via* the Wnt/β-catenin pathway is essential for appropriate bone development, and this pathway can be suppressed in response to oxidative stress, ultimately compromising bone formation (38). Vitamin C, also known as ascorbic acid, is a water-soluble vitamin that serves as a key antioxidant under physiological conditions (39). Through these antioxidant effects, vitamin C can protect against osteoporosis (40). Bone marrow stromal cells (BMSCs) can also take up vitamin C, promoting their osteoblastic differentiation in a manner that promotes bone formation (41). Sodium-coupled ascorbic acid transporter 2 (SVCT2) mediates BMSC uptake of vitamin C in a sodium level-dependent manner (42). Low-normal sodium levels have the potential to contribute to the exacerbation of oxidative stress and the suppression of bone formation through the inhibition of vitamin C uptake. Close links between inflammation and oxidative stress have been reported in a range of pathophysiological settings (43). Fibbi B et al. showed that low sodium concentrations might promote human mesenchymal stromal cells (hMSC) involvement and commitment in the adipocyte phenotype at the expense of osteoblastogenesis (44). Low-normal sodium levels may contribute to the exacerbation of inflammatory activity and oxidative stress in patients with T2D, further suppressing bone turnover.

Muscle loss may be a risk factor linked with low bone turnover in patients with T2D, given the close relationship between muscle and bone and the fact that muscle can serve as a source of myogenic stem cells that can directly play a role in bone formation (45). In animal studies, chronic hyponatremia has been linked to a range of senescence-related effects including reductions in BMD, hypogonadism, and muscle loss (46). Significant positive correlations have been detected between serum sodium levels within the normal range and the strength of upper arm muscles among older males (35). Normal serum sodium levels in older adults are also reportedly positively correlated with handgrip strength, which is commonly used as a readout for muscle strength (47). A 2019 review found that elevated fracture risk observed in patients with T2D may be in part a consequence of diabetic peripheral neuropathy (DPN) (48). Another cross-sectional study of Chinese patients with T2D detected an independent association between low-normal serum sodium levels and DPN incidence (49). Accordingly, low-normal serum sodium levels may contribute to muscle loss, neuropathy, and decreased bone turnover in patients with T2D.

In line with prior results (28, 50), no significant association between serum sodium levels and BMD was observed in this study. This may be attributable to the inability of BMD values to accurately recapitulate bone changes in patients with T2D (2). A review published in Nature Reviews Endocrinology revealed that the association between hyponatremia and fracture was independent of decreased BMD, leading to the speculation that hyponatremia might affect bone microstructure changes that is not captured by BMD (51). These results emphasized the importance of exploring the relationship between serum sodium levels and bone turnover.

There are certain limitations to this analysis. For one, this was a cross-sectional analysis, precluding the ability to detect causal relationships between sodium levels and bone turnover. In addition, analyzing serum sodium concentrations at a single time point cannot provide comprehensive insight regarding dynamic changes in these sodium levels in the body. As such, further analyses should be conducted based on sodium intake records, repeated testing of serum sodium levels, and the monitoring of urine sodium excretion. Third, this study only examined the relationship between serum sodium levels, BTMs, and BMD such that more comprehensive testing will be necessary to explore changes in the microstructural properties of bones in patients with T2D using high-resolution peripheral quantitative computed tomography (HRpQCT) or related techniques to confirm these results. Finally, all patients included in this study were Chinese, potentially limiting the generalizability of these results.

In conclusion, the present results indicate that low-normal serum levels were associated with the suppression of bone turnover in patients with T2D. This suggests that in patients with T2D, maintaining serum sodium levels within the high end of the normal range may contribute to improved bone turnover through the enhancement of bone formation, although additional *in vitro* and *in vivo* confirmation of these results will be necessary.

## Data availability statement

The original contributions presented in the study are included in the article/**Supplementary Material**. Further inquiries can be directed to the corresponding authors.

## Ethics statement

The studies involving human participants were reviewed and approved by the medical research ethics committee of the Second Affiliated Hospital of Nantong University. The patients/participants provided their written informed consent to participate in this study.

## Author contributions

H-yH and Z-qH participated in the design of the study, data collection, analysis of the data, and drafting of the manuscript. C-fL, J-bS, and X-qW conceived of the study, participated in its design and revised the manuscript. L-yH, W-sL, FX, and X-qG participated in data collection. All authors read and approved the final manuscript.

## Funding

The study was supported by the Medical Research Project of Health Commission of Nantong (MB2020012) and the Science and Technology Support Program of Nantong (HS2020005).

## Conflict of interest

The authors declare that the research was conducted in the absence of any commercial or financial relationships that could be construed as a potential conflict of interest.

## Publisher’s note

All claims expressed in this article are solely those of the authors and do not necessarily represent those of their affiliated organizations, or those of the publisher, the editors and the reviewers. Any product that may be evaluated in this article, or claim that may be made by its manufacturer, is not guaranteed or endorsed by the publisher.
