# Corrigendum: The association between normal serum sodium levels and bone turnover in patients with type 2 diabetes

**DOI:** 10.3389/fendo.2022.1088810

**Published:** 2022-11-25

**Authors:** Hai-yan Huang, Zhi-qi Huang, Ling-yan Hua, Wang-shu Liu, Feng Xu, Xiao-qin Ge, Chun-feng Lu, Jian-bin Su, Xue-qin Wang

**Affiliations:** ^1^Department of Endocrinology, Affiliated Hospital 2 of Nantong University and First People’s Hospital of Nantong City, Nantong, China; ^2^Department of General Surgery, Affiliated Hospital 2 of Nantong University and First People’s Hospital of Nantong City, Nantong, China; ^3^Department of Ophthalmology, Affiliated Hospital 2 of Nantong University and First People’s Hospital of Nantong City, Nantong, China

**Keywords:** type 2 diabetes, bone turnover, bone formation, bone resorption, sodium, bone mineral density

In the published article, there was an error in **Table 3** as published. We made a mistake in the title of the table when revising the manuscript. It was an oversight on our part that we typed the same “β” and “p” values for CTx model 0 when we made this table. The corrected **Table 3** and its amended caption “Independent associations of serum sodium level with BTMs levels via multivariate linear regression analysis” appear below.

**Table 3:** Independent associations of serum sodium level with BTMs levels *via* multivariate linear regression analysis.

Models	B (95% CI)	*β*	*t*	*p*	*R^2^* for model
OC					
Model 0	0.038 (0.020-0.056)	0.210	4.141	<0.001	0.044
Model 1	0.040 (0.022-0.059)	0.222	4.249	<0.001	0.086
Model 2	0.037 (0.019-0.055)	0.204	3.957	<0.001	0.169
Model 3	0.025 (0.003-0.047)	0.134	2.281	0.023	0.227
CTx					
Model 0	0.023 (-0.002-0.058)	0.092	1.777	0.076	0.008
Model 1	0.031 (0.006-0.057)	0.124	2.391	0.017	0.096
Model 2	0.031 (0.005-0.058)	0.125	2.362	0.019	0.128
Model 3	0.019 (-0.011-0.050)	0.074	1.247	0.213	0.212
PINP					
Model 0	0.038 (0.019-0.058)	0.196	3.854	<0.001	0.039
Model 1	0.043 (0.024-0.063)	0.224	4.409	<0.001	0.133
Model 2	0.041 (0.022-0.061)	0.214	4.150	<0.001	0.169
Model 3	0.036 (0.013-0.059)	0.179	3.023	0.003	0.215

In the published article, there also was an error in **Figure 4** as published. We made the modifications on the same template when drawing the figures, but we missed the need to modify the p value. The corrected **Figure 4** and its caption “The relationship between serum sodium and lnPINP in patientswith T2D (A) unadjusted; (B) partially adjusted for age, sex, BMIand HbA1c.” appear below.

**Figure 4:**
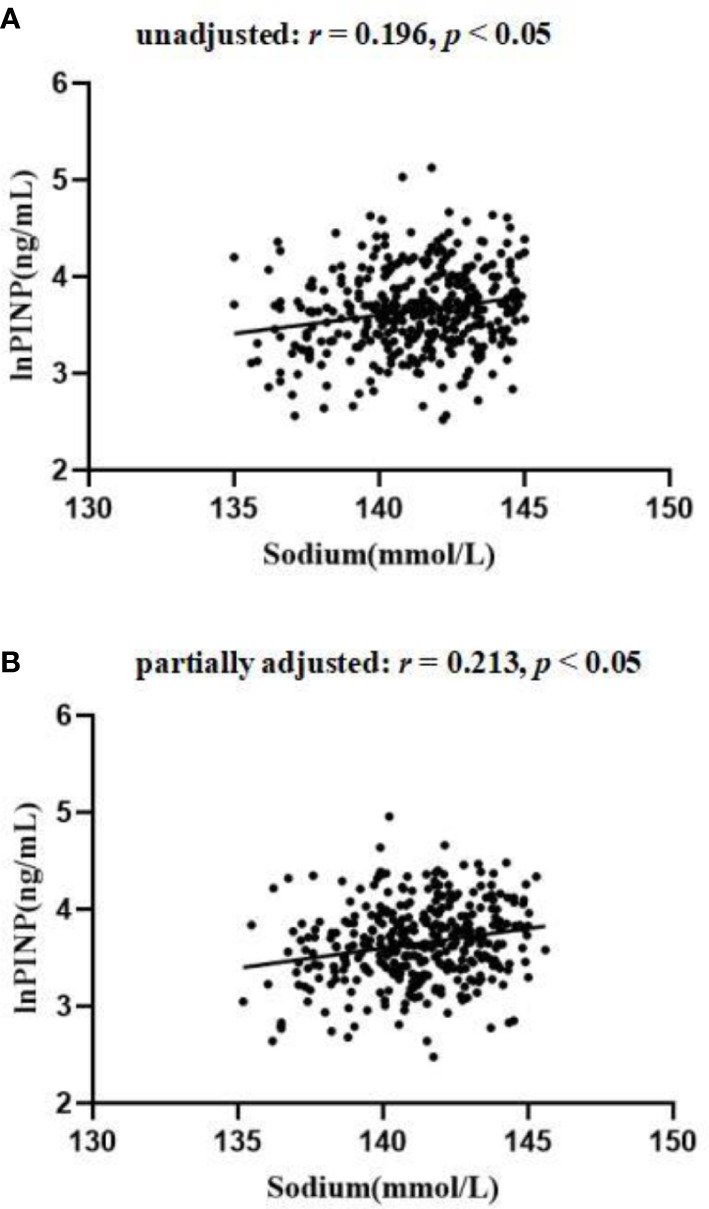
The relationship between serum sodium and lnPINP in patients with T2D **(A)** unadjusted; **(B)** partially adjusted for age, sex, BMI and HbA1c.

The authors apologize for these errors and state that they do not change the scientific conclusions of the article in any way. The original article has been updated.

## Publisher’s note

All claims expressed in this article are solely those of the authors and do not necessarily represent those of their affiliated organizations, or those of the publisher, the editors and the reviewers. Any product that may be evaluated in this article, or claim that may be made by its manufacturer, is not guaranteed or endorsed by the publisher.

