# Supplementary material for: The association between normal serum sodium levels and bone turnover in patients with type 2 diabetes
Source: Front Endocrinol (Lausanne). 2022 Oct 27;13:927223. doi: 10.3389/fendo.2022.927223 (PMC9646934; doi:10.3389/fendo.2022.927223)
Supplement: Supplementary file 1 [file Table_1.docx]

**Supplementary Table 1 Relationships between serum sodium level and clinical parameters**

| Variables | *r* | *p* value |
| --- | --- | --- |
| Age | 0.046 | 0.374 |
| Sex | 0.054 | 0.302 |
| Diabetic duration | 0.102 | 0.055 |
| BMI | 0.009 | 0.662 |
| SBP | 0.104 | 0.045 |
| DBP | -0.002 | 0.977 |
| HbA1c | -0.198 | <0.001 |
| AUC_CP_ | 0.130 | 0.027 |
| HOMA-IR_CP_ | -0.017 | 0.754 |
| Serum UA | -0.041 | 0.432 |
| Cystatin C | 0.007 | 0.903 |
| eGFR | -0.037 | 0.509 |
| UACR | -0.122 | 0.021 |
| TG | -0.131 | 0.012 |
| TC | -0.081 | 0.118 |
| HDL-c | 0.019 | 0.716 |
| LDL-c | 0.018 | 0.725 |
| Lumbar spine BMD | -0.035 | 0.573 |
| Total hip BMD | -0.043 | 0.513 |

*r* Spearman’s correlation coefficient
