# Supplementary material for: The association between normal serum sodium levels and bone turnover in patients with type 2 diabetes
Source: Front Endocrinol (Lausanne). 2022 Oct 27;13:927223. doi: 10.3389/fendo.2022.927223 (PMC9646934; doi:10.3389/fendo.2022.927223)
Supplement: Supplementary file 2 [file Table_2.docx]

**Supplementary Table 2 Relationships between serum sodium and BTMs** in elderly and non-elderly patients and in male and female patients

| Variables | Total | | Male | | Female | | Non-elderly | | Elderly | |
| --- | --- | --- | --- | --- | --- | --- | --- | --- | --- | --- |
| *n* | 372 | | 202 | | 170 | | 218 | | 154 | |
|  | *r* | *p* value | *r* | *p* value | *r* | *p* value | *r* | *p* value | *r* | *p* value |
| lnOC | 0.210 | <0.001 | 0.181 | 0.010 | 0.239 | 0.002 | 0.277 | <0.001 | 0.136 | 0.092 |
| lnCTx | 0.092 | 0.076 | 0.069 | 0.329 | 0.110 | 0.154 | 0.176 | 0.009 | -0.010 | 0.900 |
| lnPINP | 0.196 | <0.001 | 0.226 | 0.001 | 0.151 | 0.047 | 0.217 | 0.001 | 0.179 | 0.029 |
